# Glutathionylspermidine in the Modification of Protein SH Groups: The Enzymology and Its Application to Study Protein Glutathionylation

**DOI:** 10.3390/molecules20011452

**Published:** 2015-01-15

**Authors:** Jason Ching-Yao Lin, Bing-Yu Chiang, Chi-Chi Chou, Tzu-Chieh Chen, Yi-Ju Chen, Yu-Ju Chen, Chun-Hung Lin

**Affiliations:** 1 Institute of Biological Chemistry, Academia Sinica, 128 Academia Road Section 2, Taipei 11529, Taiwan; E-Mails: jcl@gate.sinica.edu.tw (J.C.-Y.L.); bingyuchiang@gmail.com (B.-Y.C.); sandra77@gate.sinica.edu.tw (C.-C.C.); ebioche@gmail.com (T.-C.C.); 2 The Core Facilities for Protein Structural Analysis, Academia Sinica, 128 Academia Road Section 2, Taipei 11529, Taiwan; 3 Institute of Chemistry, Academia Sinica, 128 Academia Road Section 2, Taipei 11529, Taiwan; E-Mails: tp7249@gmail.com (Yi-J.C.); yjchen@chem.sinica.edu.tw (Yu-J.C.); 4 Department of Chemistry and Institute of Biochemical Sciences, National Taiwan University, Taipei 10617, Taiwan

**Keywords:** glutathione, redox, proteomics, glutathionylation, transglutaminase

## Abstract

Cysteine is very susceptible to reactive oxygen species. In response; posttranslational thiol modifications such as reversible disulfide bond formation have arisen as protective mechanisms against undesired *in vivo* cysteine oxidation. In Gram-negative bacteria a major defense mechanism against cysteine overoxidation is the formation of mixed protein disulfides with low molecular weight thiols such as glutathione and glutathionylspermidine. In this review we discuss some of the mechanistic aspects of glutathionylspermidine in prokaryotes and extend its potential use to eukaryotes in proteomics and biochemical applications through an example with tissue transglutaminase and its *S*-glutathionylation.

## 1. The Roles of Glutathione in Prokaryotes

**Figure 1:**
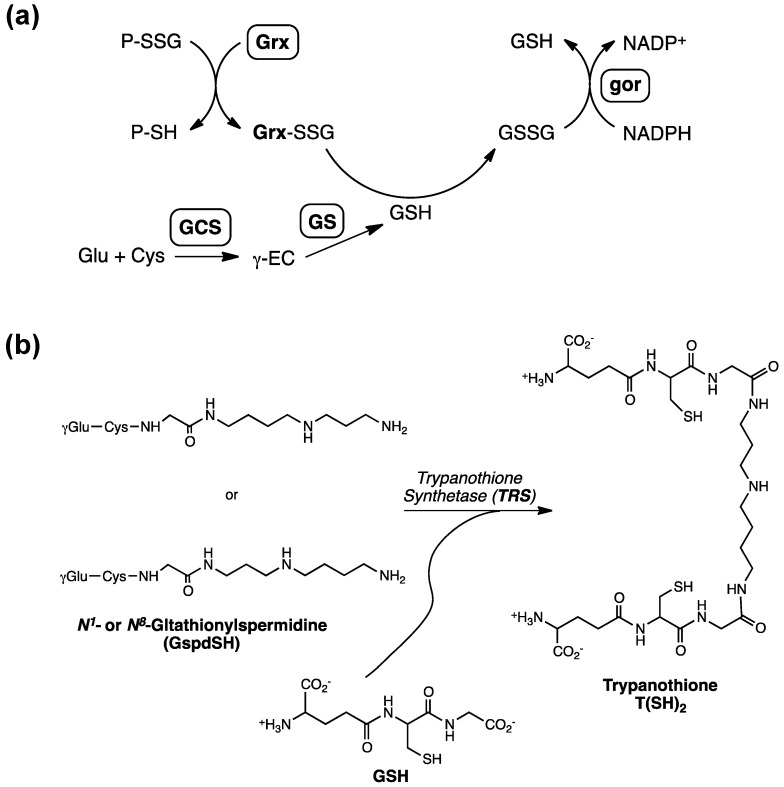
(**a**) Metabolic pathway of glutathione (GSH) involving γ-glutamylcysteine synthetase (GCS), glutathione synthetase (GS) as well as the enzymatic cycling of GSH reductase (gor) and glutaredoxin (Grx) to facilitate the conversion of GSH to GSSG. Reduction of P-SSG by Grx leads to a Grx-SSG intermediate that is further reduced by GSH to produce GSSG, which is reduced by gor (γEC: γ-glutamylcysteine). (**b**) The enzymatic steps involved in the biosynthesis of glutathionylspermidine and trypanothione [18]; Figure 1b was originally published by Tetaud *et al.* [18] © the American Society for Biochemistry and Molecular Biology.

Higher organisms commonly utilize oxidation to defend from and combat pathogenic invasions by other species. For example, NADPH oxidase and nitric oxide synthase are rapidly produced by neutrophils and phagocytes, respectively, to destroy intruding microbes once the innate immune system identifies the invaders. In phagocytes, superoxide (O_2_^−•^) is generated by the NADPH oxidase and can be subsequently converted to hydrogen peroxide (H_2_O_2_), peroxynitrite (ONOO^−^) or hypochlorous acid (HOCl), all of which are common reactive oxygen species (ROS) [1]. An evolutionary countermeasure for pathogens to evade oxidative stress within their hosts is to then develop efficient reducing agents or pathways to eliminate ROS. Catalases, for instance, efficiently catalyze the decomposition of hydrogen peroxide. The thiol-redox buffer glutathione (GSH) is a low-molecular weight (LMW) thiol that regulates intracellular thiol redox balance and protects against oxidative stress [2,3]. GSH is a tripeptide, composed of glutamate, cysteine and glycine. The thiol group of glutathione can target electrophilic conjugate acceptors for reduction. Modification of cysteine residues by GSH can prevent further irreversible sulfinate or sulfonate formation, and thus serves a protective purpose. GSH can be converted to its oxidized form, glutathione disulfide (GSSG). Several enzymes, such as GSH peroxidase and glutaredoxin, participate in redox homeostasis *in vivo*, while GSH reductase ensures that GSH remains at a reduced state and subsequently maintains a high GSH/GSSG ratio in the cytoplasm [2]. Intracellular GSH is typically maintained at 5–10 mM *in vivo* and mostly exists in a reduced form; for instance, the ratio of reduced to oxidized glutathione in *E. coli* is about 200 to 1 [4]. GSH is kept in a reduced state by glutathione reductase (gor, see Figure 1a). While GSH is present in most of eukaryotes, its production among prokaryotes is restricted to cyanobacteria, proteobacteria and a few strains of Gram-positive bacteria; others rely on similar small molecular weight thiols as substitutes for GSH, such as glutathione amide, glutamylcysteine, mycothiol and bacillithiol [5,6,7,8,9,10,11]. Most Gram-positive bacteria, e.g., Firmicutes and Actinomycetes, do not utilize GSH or cysteine as their redox buffers [12,13]. Additionally, GSH serves a protective role against external stresses including oxidative stress induced by peroxides, such as H_2_O_2_ or alkyl hydroperoxides [14] as well as toxins like methylglyoxal [15]. In *E. coli*, the biosynthesis of GSH is catalyzed by γ-glutamylcysteine synthetase (GCS) and GSH synthetase (GS) (Figure 1a), both of which, along with glutathione reductase and glutaredoxin 1 (grxA), are induced during oxidative stress [2]. While GSH does not respond to H_2_O_2_ directly [14], sensitivity to diamide, a known inducer of oxidative stress *in vivo*, is observed. It has been reported that *E. coli* gor-knockout mutants are sensitive to cumene hydroperoxide and show increased H_2_O_2_ sensitivity in a catalase mutant background [16]. Glutaredoxins (Grx) performs a critical role in the reduction of the GSH-protein mixed-disulfide (Figure 1). Most of glutaredoxins have a conserved C-P-Y-C motif, which participates in disulfide exchange [17] to reduce GSH mixed disulfides. Glutaredoxin reduces the protein-GSH mixed disulfide via the formation of a Grx-GSH intermediate which is subsequently reduced by a second molecule of GSH [16].

## 2. Glutathionylspermidine (Gsp) in *E. coli* and Protozoa

In *E. coli* and trypanosomatid parasites, GSH can be enzymatically converted to N1-glutathionyl-spermidine (Gsp) by an ATP-cleaving, amide-forming reaction with spermidine (Spd) catalyzed by Gsp synthetase [18,19]. In parasites, subsequent glutathionylation at the N^8^ of Gsp leads to N1,8-bis(glutathionyl)-spermidine, which is known as trypanothione. Gsp was first discovered in *E. coli* nearly four decades ago [20] and had been found to be superior to GSH in its reducing efficiency. Thus, the cycling between the various oxidation states of cysteine residues between the thiol state (P-SH) and a Gsp *S*-thiolated (PSSGsp) state can prevent further oxidation to irreversible states such as the formation of cysteine sulfinic or sulfonic acids. The non-enzymatic reductions of dehydroascorbate by Gsp, for instance, are several times faster than that by GSH [21]. Additionally, Gsp consumes hydrogen peroxide three times faster than GSH [22] and has been proposed to be more effective at preventing DNA damage induced by free radicals or oxidative species, owing to the high affinity of the spermidine moiety for negatively charged nucleotides [23]. Only Enterobacteria and some distantly related eukaryotic Kinetoplastida, most of which are parasites, demonstrate high sequence Gsp synthase/amidase homology [24] to suggest the existence of a Gsp system. Enterobacteria, for instance, are facultative, Gram negative anerobes. Based on BLAST results against *E. coli* Gsp synthetase/amidase (accession number AAC76024.1), other pathogens such as *Shigella flexneri* and *Shigella dysenteriae* may also utilize Gsp for maintaining redox homeostasis.

Gsp, along with other small molecular weight thiol derivatives such as trypanothione, has been implicated to be integral to oxidative defense of various pathogens [19], but no clear linkage has been established between Gsp and pathogenicity. Recently, Ansong *et al*., through a top-down proteomics approach observed potential evidence connecting protein *S*-thiolations, e.g., *S*-glutathionylation and *S*-cysteinylation in response to infection-like conditions in *Salmonella typhimurium*, a Gsp-containing Entereobacterium [25]. However, this phenomenon was not attributed to Gsp despite its presence in *S. typhimurium*. We previously reported marginal increases in the sensitivity of a combined grxD and gssD double mutant to hydrogen peroxide, but no difference between gss^+^ and gssD cells was observed [26]. At the current stage, while Gsp may potentially participate in pathogenicity, there is insufficient evidence to suggest so.

While Gsp only exists in unicellular organisms, it exists not as a precursor to the current ROS-scavenging system found in modern organisms today, but more likely to be a parallel defense strategy instead. The deletion of Gsp at the genomic level was found to cause changes in regulation of several transcriptional genes, with some related to growth in low oxygen or low pH by Tabor and colleagues [24]; global transcriptome analyses via DNA microarray curiously inferred that glutathionylspermidine was not present with clear functions for most species. The biosynthesis of Gsp requires ATP and the strict negative regulation of Gsp amidase. This system is too energetically expensive to undergo co-evolution only to be removed from the genome entirely. Since Gsp is only found in Enterobacteria, it is likely that in pathogenic species this mechanism surfaced as secondary metabolites from the production of ROS species to evade the host defense system. As spermidine may induce apoptosis via ROS generation in mammalian cells [27], Enterobacteria may exploit such a mechanism to generate excess spermidine by spermidine synthase for host invasion and subsequently Gsp to prevent autopoptosis from the resultant excess ROS. To-date, the true physiological function of Gsp remains relatively unknown. It is probable that while Gsp offered a higher redox potential than GSH, the yield is relatively marginal at best outside infectious stages. The energetic expenditure of producing Gsp solely in place of glutathione thus offers diminishing returns and thus becomes slowly phased from microbes other than Enterobacteria. Furthermore, other mechanisms of regulating the reducing capacity of GSH, such as persulfuration [28,29] may also have facilitated the elimination of Gsp altogether.

**Figure 2:**
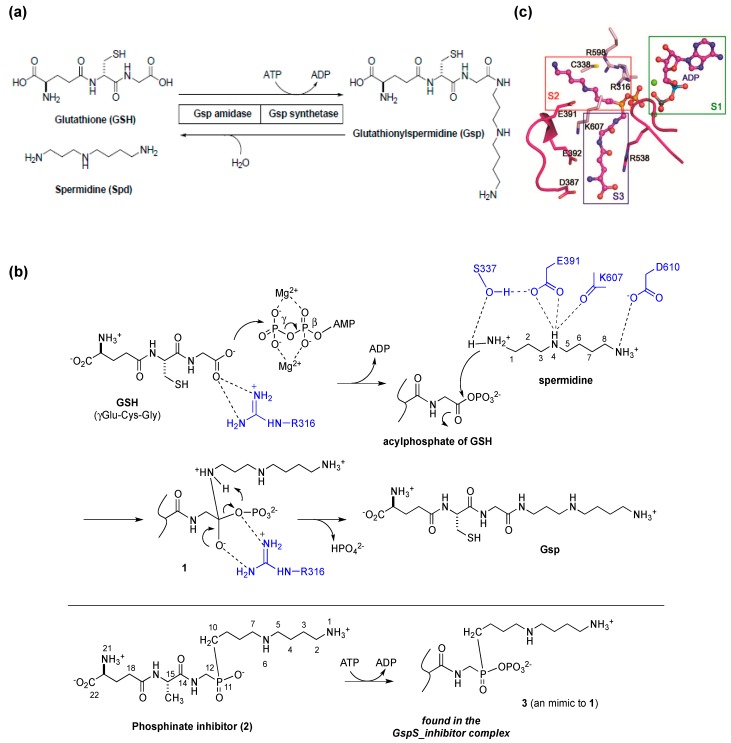
(**a**) The reactions catalyzed by *E. coli* glutathionylspermidine synthetase/amidase (GspSA). GspSA from *E. coli* is a bifunctional protein that contains an N-terminal amidase domain and a C-terminal synthetase domain. The two catalyzed reactions are shown at the molecular level; (**b**) Proposed reaction mechanism of GspS. The conjugation reaction of GSH with spermidine proceeds in two steps—the C terminus of GSH is initially phosphorylated by γ-phosphate of ATP to form an acylphosphate, followed by the nucleophilic attack of N1-spermidine to the acylphosphate; (**c**) Structure of inhibitor-bound GspS (*GspS_inhibitor*) at the S2 and S3 substrate-binding pockets near four negatively charged residues Asp387, Asp389, Glu391 and Glu392. Figure 2a was reproduced from [26] © The American Society for Biochemistry and Molecular Biology. Figure 2b,c were reproduced from [30] © European Molecular Biology Organization.

In 1995, Walsh et al identified the sequence of Gsp synthetase, and first purified the enzyme, which was surprisingly found to be also capable of hydrolyzing Gsp to GSH and spermidine [19]. The bifunctional Gsp synthetase/amidase (GspSA) has two separate activity domains, a *N*-terminal amidase and a synthetase domain near the C-terminus. (Figure 2a).

Based on the enzymatic assay of spermidine analogues, the same research team proposed that the catalytic mechanism of Gsp synthetase is similar to the enzymes catalyzing the ATP-dependant amide-bond formation, such as glutamine synthetase and glutathione synthetase. The inhibition of Gsp synthetase by Gsp-phosphonate and -phosphinate analogues supported the idea that N1 of spermidine acts as the nucleophile to attack the acylphosphate intermediate. In 2006, Lin, Wang and coworkers successfully resolved several X-ray crystal structures of *E. coli* GspSA in complex with various ligands [30]. These structures both clarified the synthetase reaction mechanism, and represented the first step in understanding the structural and functional differences within the enzyme family. The conjugation reaction of GSH with spermidine proceeds in two steps—the C-terminus of GSH is initially phosphorylated by ATP to form an acylphosphate before a nucleophilic attack of N1-spermidine. The resulting tetrahedral adduct then collapses to form an amide bond and breaks the C–O bond of the phosphate, leading to the formation of Gsp and release of inorganic phosphate, respectively, with ADP also being released after catalysis (Figure 2b). The side chain of GspS Glu391 holds a key role in spermidine binding, as is Ser337 to facilitate the nucleophilic attack during catalysis by forming H-bond with N1-amine (Figure 2c). Interestingly, GSH appears to bind at two different sites, suggesting that the synthetase domain operates by translocation catalysis. GSH is phosphorylated at one GSH-binding site to form an acylphosphate intermediate, and the intermediate is then translocated to the other binding pocket before the subsequent nucleophilic addition of spermidine.

Iodoacetamide (IAM) inactivation and site-direct mutagenesis identified the catalytic residues of Gsp amidase as Cys 59 [31]. Multiple sequence alignment showed that Gsp amidase belongs to the CHAP domain superfamily (cysteine, histidine-dependent amidohydrolases/peptidases). Intriguingly, the hydrolytic activity of the Gsp amidase domain alone is 10-fold higher than that of full length GspSA [31]. The mechanism in which the synthetase domain dictates amidase activity through interdomain communication has yet to be fully elucidated at molecular basis, but it has been inferred that an *in vivo* steady-state ratio of substrate and product may be critical in maintaining homeostasis [31]. Interestingly, if Gsp is involved in redox regulation or oxidative defense in *E. coli*, it will be necessary to determine the process and related enzymes responsible for reducing the oxidized form of Gsp disulfide. As yet, there have been no reports indicating the existence of a Gsp-specific reductase in *E. coli*.

## 3. Posttranslational Thiol Modifications of Cysteine

**Figure 3:**
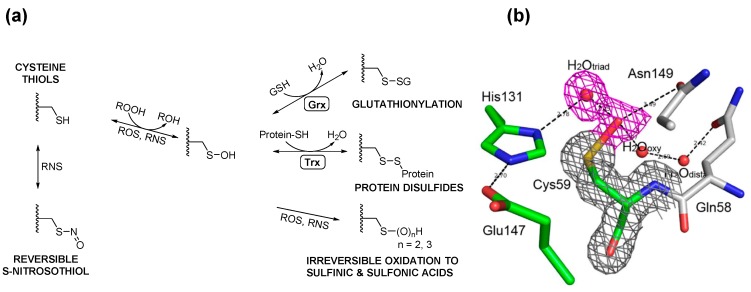

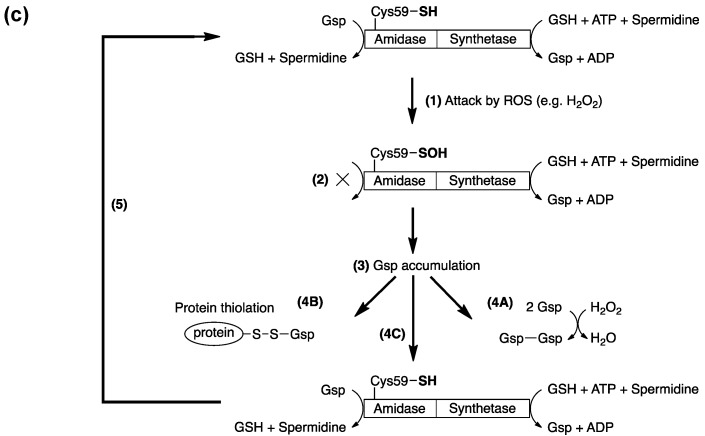
(**a**) Cysteine thiols can be oxidized by different oxidants to form sulfenic acid, which may be stabilized or go on to form various reversible or irreversible species. The thiol moiety may be reversibly oxidized to form cysteine sulfenic acid or *S*-nitrosothiol by reactive oxygen (ROS) or nitrogen (RNS) species, respectively. Sulfenic acids may be reduced to mixed disulfides with glutathione or proteins upon reduction (R-SOH to R-SH and the formation of GSSG) and further disulfide exchange of R-SH with GSSG to R-SSG. These disulfides are enzymatically reversible in the governance of enzymes such as glutaredoxin (Grx) and thioredoxins (Trx). ROS and RNS may further oxidize cysteine sulfenic acids irreversibly to sulfinic and sulfonic acids as well; (**b**) View of the 2*Fo–Fc* electron density map at the active site of H_2_O_2_-treated GspA. The electron density map, drawn at a contour level of 1σ, shows continuous density (in *red*) connected to the Sγ atom of Cys59. This density was fitted with the oxygen atom of a sulfenic acid (R-SOH) and the oxygen of a tightly H-bonded water molecule. The corresponding H-bond is very short (2.2 Å); (**c**) *E. coli* maintains intracellular redox homeostasis through a Gsp *S*-thiolation cycle. Upon exposure to ROS, the active-site thiol (Cys59) of Gsp amidase is oxidized (1) to sulfenic acid, rendering the inactivation (indicated by ×) of Gsp amidase (2); however, since the synthetase domain is unaffected, the concentration of intracellular Gsp begins to accumulate (3), which drives the formation of Gsp- or other small molecule disulfides to protecting protein thiols from over-oxidation (4A, 4B). Upon homeostasis, Gsp amidase is reduced by intracellular GSH or Gsp, which leads to the restoration of its activity (4C). Reactivated Gsp amidase may hydrolyze Gsp or hydrolytically remove Spd from Gsp-disulfide or Gsp-modified proteins to return the Gsp concentration to its basal level (5). A more detailed description of this current model can be found in [26]. Figure 3b,c were originally published by Chiang *et al*. [26] © The American Society for Biochemistry and Molecular Biology.

ROS such as H_2_O_2_, HOCl, organic hydroperoxides and peroxynitride often indiscriminately attacks various biomolecules including DNA, proteins and lipids, and subsequently causes cell damage. Components most susceptible to oxidative damage are proteins, of which ROS often react with cysteine and tyrosine side chains to produce oxidized adducts. Cysteine residues involving in enzyme catalysis or functional regulation are more reactive since the sulfhydryl groups have lower pK_a_ values resulting in deprotonated thiolate anions. These anions are prone to oxidation by ROS to form sulfenic acid (R-SOH) intermediates. Such intermediates are further reduced to reversible protein disulfides (intramolecular and intermolecular protein disulfides or mixed protein disulfides with LMW thiols), in a phenomenon known as protein *S*-thiolation. In the absence of proximal thiols, the sulfenic acid intermediate may be irreversibly oxidized (Figure 3a) to cysteine sulfinic (R-SO_2_H) or sulfonic acids (R-SO_3_H) [32,33]. The “over-oxidation states” of the thiol group, including sulfinic acid and sulfonic acid, have been considered biologically irreversible. Nonetheless, recent evidence suggests that reduction of the sulfinic acid form of some human peroxyredoxins can occur *in vivo* [34]. The irreversible oxidation for reactive cysteine may lead to protein dysfunction. Therefore, a mechanism is necessary to prevent irreversible oxidation *in vivo*. For this purpose, protein *S*-glutathionylation (PSSG) exists as one of the mechanisms to alleviate protein over-oxidation. Intriguingly, a major component of the ROS-linked modulation of cell signaling pathways is the dynamic regulation of protein function by reversible thiol modification.

For instance, increased levels of tyrosine phosphorylation mediated by ROS suggest that their effects are exerted via inhibition of protein tyrosine phosphatases (PTPs). Reactive oxygen species have been reported to transiently inactivate PTPs via a reversible oxidation reaction of their catalytic cysteines at low concentrations of hydrogen peroxide (<50 μM) [35,36].

## 4. Protein *S*-Thiolation with GSH and Gsp

Protein *S*-thiolation, the formation of mixed disulfides between cysteine residues and other thiols such as glutathione and cysteine, is one of the many posttranslational modifications involved in biological events. The formation of mixed disulfides occur through several different mechanisms, such as thiol/disulfide exchange reactions between a protein sulfhydryl group and GSSG, or the oxidation of the protein thiol to sulfenic acid followed by a subsequent reaction with GSH (Figure 3a). Additionally, *S*-glutathionylation can arise from the reactions between GSH and *S*-nitrosylated cysteines or between cysteine thiols and *S*-nitrosothiols such as GSNO.

Protein *S*-glutathionylation is a protective mechanism against oxidative damage as well as a regulatory mechanism of enzyme activities. PSSG also participates in cell energy metabolism and signaling pathway. Several glycolytic enzymes, such as aldolase, pyruvate kinase and phosphoglycerate kinase are glutathionylated in rat or human hepatocytes when exposing to oxidative condition [37]. Studies also indicated the role of PSSG in the regulation of PTP1B and MEKK1 in response to oxidative stress [38,39]. Moreover, cytoskeletal assembly and calcium homeostasis were also found to be regulated by PSSG [40,41]. PSSG could also be observed in processes such as inflammation, proliferation, differentiation and apoptosis through targets such as STAT3, eNOS, the 20S proteasome and histone S3 [42,43,44,45,46].

GspSA is essential for controlling Gsp levels in response to redox conditions. We proposed a GspSA-based model [26] to explain its role in redox response. The transient inactivation of Gsp amidase led Gsp to accumulate in response to oxidative stress, and the coupling of Gsp amidase and GSH reductase converted oxidized forms of Gsp (Gsp-disulfide and other mixed disulfides) to GSH (Figure 3c). The active-site nucleophile of Gsp amidase, Cys59, was found to yield a cysteine-sulfenic acid in the presence of H_2_O_2_ by X-ray crystallography, chemical modification by dimedone, and subsequent mass spectrometric analysis. The sulfenic acid could be reduced by addition of thiols to restore the amidase activity. Despite this highly reactive feature, the sulfenic acid moiety is stabilized by formation of several hydrogen bonds (H-bonds), one of which is unusually very short (2.2 Å, shown in Figure 3b). We also found that in *E. coli* Gsp also modified proteins in a similar manner as PSSG in mammalian cells. The role of GspSA in oxidative defense is favored by the hypersensitivity of H_2_O_2_-treated gspSA-/grxA- null mutants. Additionally, since GspSA has two opposing catalytic activities, the selective inactivation of amidase activity by H_2_O_2_ could result in the accumulation of Gsp, which was observed and analyzed by HPLC chromatograms of monobromobimane-derived thiol compounds [47]. In particular, the Gsp level was four times higher than that of GSH when H_2_O_2_ was present, compared to a marginal increase in the absence of H_2_O_2_.

**Figure 4:**
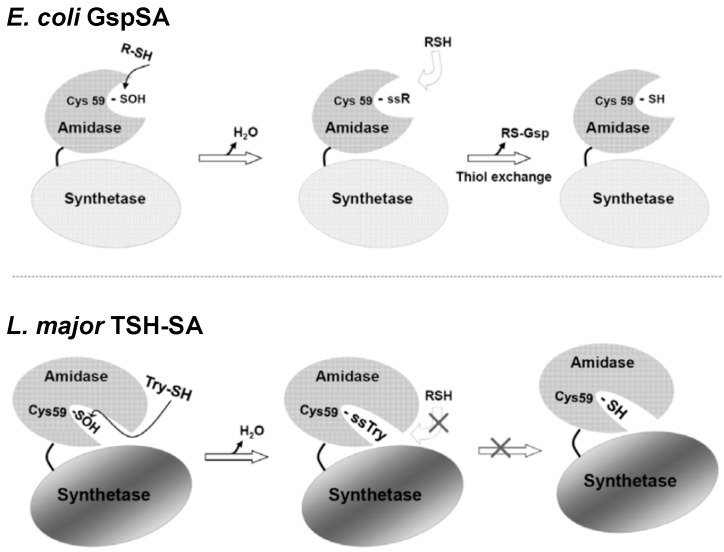
Schematics illustrating the different redox regulation mechanisms for *E. coli.* GspSA and parasitic protozoa TSH-SA [26]. The recovery of Gsp amidase activity in *E. coli* may occur via nucleophilic attack by Gsp or GSH (or other small thiol compounds) to form a cysteine-mixed disulfide. Because Cys59 is accessible to solvent, the disulfide can then be reduced via thiol-exchange with a GSH or Gsp. Conversely, the TSH amidase substrate-binding channel is narrow and solvent inaccessible, because it is blocked by the C terminal region of the synthetase domain (residues 634~652) and cannot accommodate the presence of two TSH molecules for disulfide exchange. This research was originally published by Chiang *et al*. [26] © The American Society for Biochemistry and Molecular Biology.

Once an oxidative stress is eliminated, the inactivated Gsp amidase activity could be rescued by reaction with GSH or Gsp, the latter of which accumulates during the stress. Sulfenic acid is a reactive electrophile and likely reacts with thiol reagents such as GSH to generate a mixed disulfide. The location of the Gsp amidase binding site [48] on the protein surface allows free access of GSH or other thiol reagents to the Cys59-sulfenic acid. Once a mixed disulfide is formed on Cys59, thiol exchange may continue, producing the free thiol and recovering the Gsp amidase activity (Figure 4). The reactivated amidase may then hydrolyze excessive Gsp back to GSH and spermidine.

## 5. Potential Biological Applications

**Figure 5:**
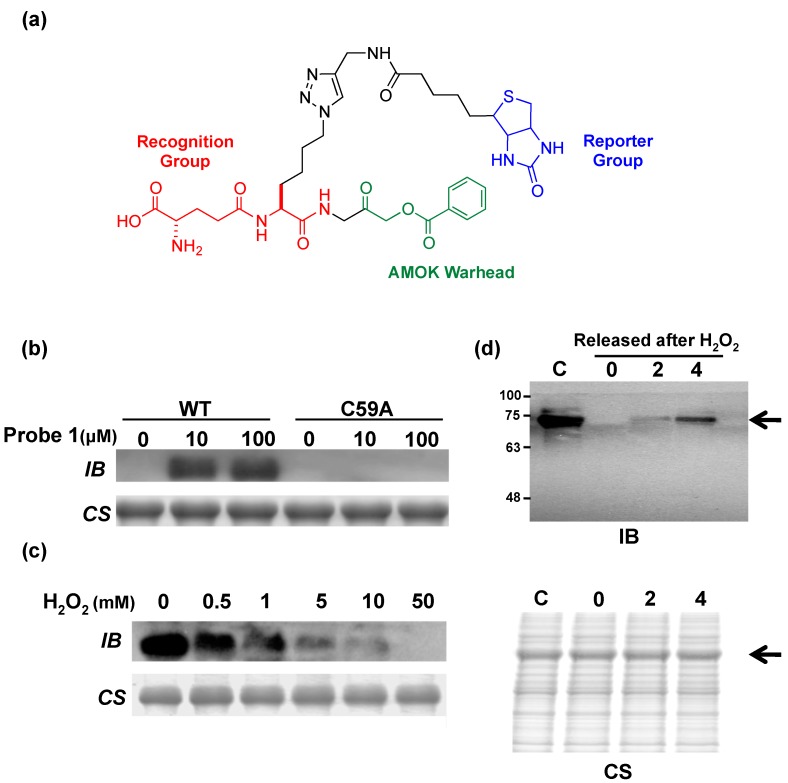
(**a**) Structure of an acyloxymethyl ketone (AOMK) activity-based probe (ABP) for the characterization of Gsp amidase in *E. coli* [49]. A typical ABP includes a warhead for covalent crosslinking (in color green), a domain to enhance binding selectivity (red), and a reporter group to allow for experimental characterization (blue); (**b**) Recombinant Gsp synthetase/amidase (GspSA, “WT”) and the mutant C59A could be distinguished by immunoblotting; (**c**) The extent of GspSA deactivation by H_2_O_2_ could be differentially assayed; (**d**) GspSA could be specifically identified *in vivo*. Upon peroxide treatment, the amount of active Gsp amidase in *E. coli* cultures could be measured. The arrow identifies the expected position of GspSA (MW of 70 kDa). WT: wild-type GspSA; IB: immunoblotting using anti-biotin; CS: Coomassie Blue staining; C: negative control, no H_2_O_2_ treatment. Lanes marked 0, 2, 4, denote the duration of recovery (h) after H_2_O_2_ release [49]. © Wiley-VCH.

GspSA has been found in a number of species based on DNA sequence alignment; these organisms include *Salmonella enterica*, *Klebsiella pneumoniae* and *Shigella flexneri*. One may reasonably postulate that GspSA in these species may serve similar functions due to their similar nature and environment in pathogenic species, e.g., pathogen defense mechanisms. Since GspA belongs to the CHAP domain superfamily with mechanistically similar catalytic residues, activity-based probes with an acyloxymethyl ketone (AOMK) warhead (Figure 5a) [49] can be designed or applied to elucidate the mechanisms behind the evasion of immunological systems. Activity-based probes (ABP) are chemical constructs often extended from inhibitor designs; a reactive group (or “warhead”) covalently links the ligand directly onto the catalytic nucleophilic residue, while a recognition domain on the probe enhances selectivity and a reporter group provides mechanisms for visualization or affinity purification of the labeled enzyme. ABPs allow the labeling of active enzymes and has the ability to discriminate inactive enzymatic species such as zymogens or inhibitor-bound enzymes [50]. With instrumentation such as mass spectrometry [50] or fluorescence imaging [51], labeled enzymes can be detected and identified, allowing for targeted biochemical studies or proteomic profiling. The AOMK-infused activity-based probe specifically targeted Cys59 (Figure 5b) and could distinguish between active and deactivated (by H_2_O_2_) GspA in a dose-dependent manner (Figure 5c). The probe also exhibited excellent specificity *in vivo* (Figure 5d). Results also echoed observations from the previous model that GspA under physiological conditions underwent reversible ROS- or RNS-induced inactivation, which was gradually restorable over time. Aside from understanding the role of GspSA in redox regulation in *E. coli*, the use of such a probe could as well be extended to elucidate the physiological function of Gsp *in vivo*.

A possibility of utilizing the enzymatic mechanism of Gsp is to probe PSSG based on its ability to derivatize endogenous GSH *in vivo*. Conventional methods to detect PSSG typically depend on the use of radiolabeled cysteines, anti-GSH antibodies or biotin switches, all of which have shortcomings and technical hurdles for further applications. For instance, the labeling of endogenous GSH with radioactive 35S-cysteine, followed by phosphor-imaging on 2D-PAGE [37], does not distinguish different types of *S*-thiolation, e.g., cysteinylation from glutathionylation. Most of the aforementioned methods are also short of straightforward procedures to enrich and identify these modified proteins [52]. Commercially available anti-GSH antibodies can be used, but serious concerns regarding their specificity and sensitivity have also been raised [52]. With advances in mass spectrometry, newer analytical methods such as glutaredoxin-dependent biotin-switch methods have been developed [53]; however, the requirement of alkylation of reduced cysteine residues as well as further reduction of GSH-modified cysteines with bacterial glutaredoxin and subsequent biotin labeling can introduce serious analytical complications such as incomplete reduction/alkylation, poor bacterial glutaredoxin specificity, and unintentional cysteine oxidations due to ambient exposure. Biotinylated GSH disulfide (biotin-GSSG) has also been used to directly label protein cysteines [54], but the external addition of biotin-GSSG likely alters the normal thiol content and the GSH/GSSG ratio given that GSSG usually accounts for less than 1% of total GSH in mammalian cells [55]. Furthermore, since GSSG is not a major route of PSSG under physiological conditions, such a method may exclude an unexpectedly large pool of proteins during analysis.

**Figure 6:**
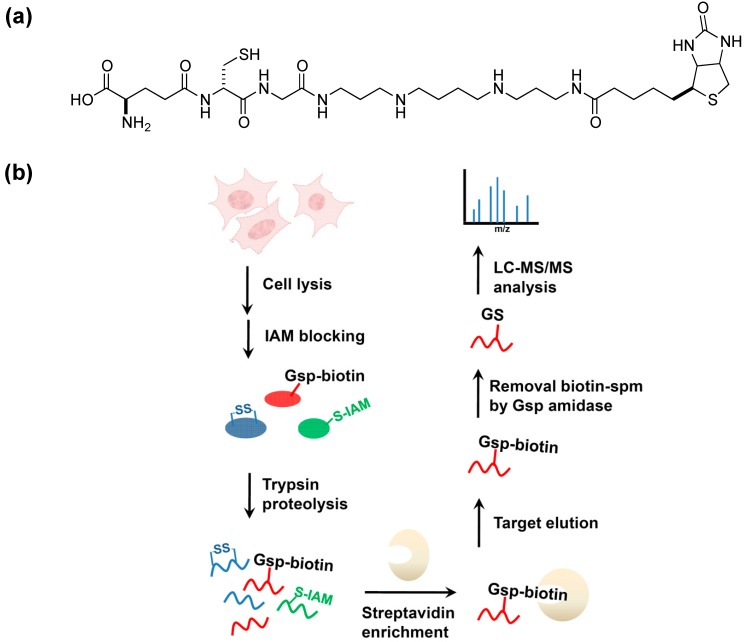

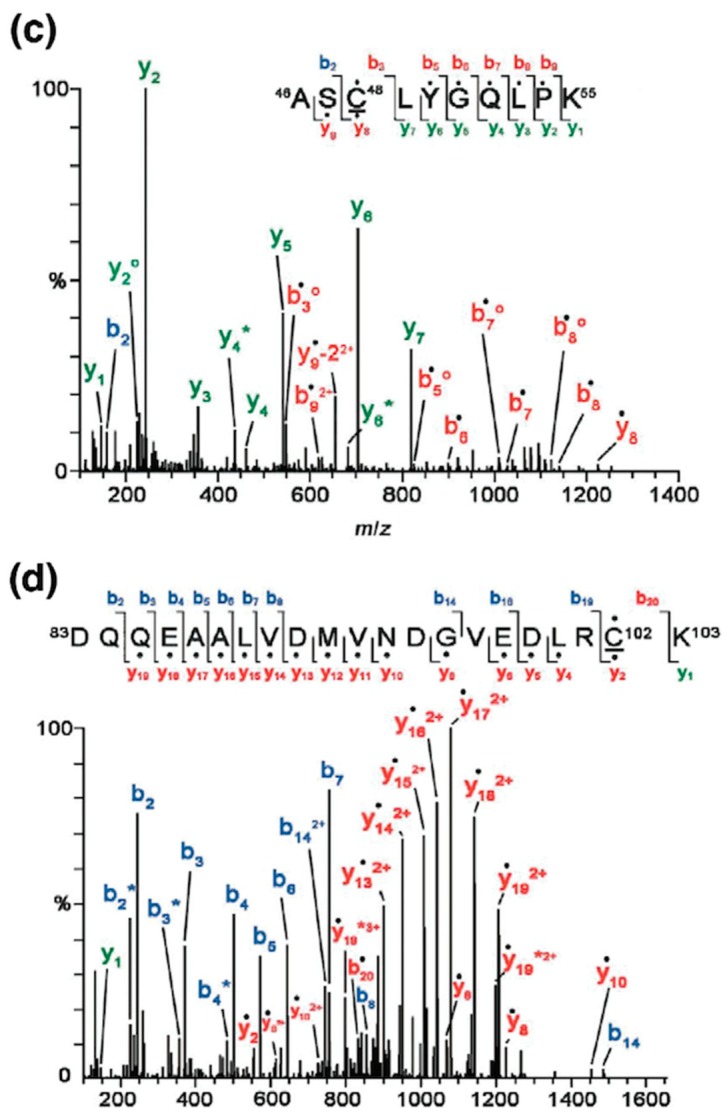
GspS as a tool for probing protein *S*-glutathionylation [56]. (**a**) the structure of biotinylated glutathionylspermine, Gsp-biotin. This substrate is readily accepted by Gsp amidase and can be hydrolyzed to GSH and spm-biotin enzymatically; (**b**) Workflow for site-specific identification of protein glutathionylation in *gsps*-transfected 293T cells. Cells are lysed and treated with iodoacetamide (IAM) to block free thiols. After tryptic digestion, Gsp-biotin *S*-thiolated peptides are enriched by streptavidin resin, followed by GspA hydrolysis to remove biotin-spm, leaving intact GSH on labeled peptides; (**c**,**d**) are MS/MS spectra derived from two glutathionylated tryptic peptides of glutathione *S*-transferase pi (GSTπ), which afford the [M+2H]^2+^ precursor ion at *m/z* 692.82 for the Cys48-containing peptide (c) and the [M+3H]^3+^ precursor ion at *m/z* 885.05 for the Cys102-carrying peptide (d). The amino acid sequences and respective b- and y- ions are shown in each spectrum, with the glutathionylated cysteine residues underlined. Filled circles (dots) above the labeled ions indicate product ions carrying a GSH moiety, which contributes to a mass increment of 305 Da relative to those ions without modification. Open circles (O) and asterisks (*) indicate product ions containing a neutral loss of H_2_O and NH_3_, respectively. “‒2” on y-ions indicates the presence of cysteine thioaldehyde residues, which are 2 Da lower than cysteine in the molecular weight [56] © Wiley-VCH, reproduced with modifications in colors and lettering.

We recently developed a method [56] for profiling PSSG in mammalian cells by using biotinyl-spermine (biotin-spm) and *E. coli* glutathionylspermidine synthetase (GspS) as a potential solution to this difficult problem. The catalysis of amine-bond formation between GSH and spermidine by GspS and the previous discovery that Gsp behaves similarly to GSH in forming disulfide bonds with cysteine residues of proteins *in vivo* make this a potential application for GspSA. By expressing GspS in mammalian cells, the resultant mixed-disulfide bond formation between Gsp and protein cysteines is equivalent to in-situ labeling of PSSG with spermidine. The introduction of pCMV2B-GspS into human embryonic kidney (HEK) 293T cells allowed for the expression of GspS and the subsequent conversion of endogenous GSH to Gsp *in vivo*. Crystal structures of GspS-substrate complex suggested that the introduction of a biotin moiety to N10 position of spemidine protruded into the solvent and thus did not participate in binding [30]. As such, chain lengthening and further derivitization beyond this location hypothetically should not interfere with enzyme activity. When spermine biotinylated at one of its terminal amines is used in place of spermidine, Michaelis-Menten kinetics parameters indicate that biotin-spm (K_m_ and k_cat_ of 74 μM and 2.7 s^−1^, respectively) is as comparable as the native substrate spermidine [30]. The morphology of viable 293T cells and MTT (3-(4,5-dimethylthiazol-2-yl)-2,5-diphenyl tetrazolium bromide) assay indicated that biotin-spm does not dramatically alter cell viability. Therefore, neither biotin-spm nor biotinylated Gsp (Gsp-biotin, the enzyme reaction product; Figure 6a) caused considerable cytotoxicity.

To identify glutathionylated proteins and their modified cysteines, we developed a workflow (Figure 6b) and identified 1409 unique glutathionylated cysteines from 913 proteins that were found to undergo PSSG. Results included candidates such as glutathione *S*-transferase pi (GSTπ) and sarco/endoplasmic reticulum Ca^2+^-ATPase (SERCA) [41,57,58] (Figure 6c,d), as well as several others [59,60,61,62,63,64,65,66,67]. The use of such a method prevents the alteration of intracellular thiol content and complex experimental steps typically associated with biotin switches, as well as the issue of ion supression in mass spectrometry when biotin is introduced in the system. Particularly, the enzymatic labeling GSH by GspS can distinguish the intramolecular (protein disulfide bonds) and different types of intermolecular disulfide linkages (glutathionylation and cysteinylation). None of the available methods are able to detect the attached GSH to provide definitive evidence of site-specific glutathionylation [52]; in addition, the fragmentation efficiency of biotin tag is often too low to reveal site-specific information in the MS/MS spectrum [68] to account for neutral losses caused by collision-induced fragmentation [69]. In the human proteome, protein GSH *S*-thiolation is functionally different from other modes of *S*-thiolation, e.g., *S*-cysteinylation; as such, the development and utilization of a separate set of characterization methods is necessary to discern said differences. For instance, GSH *S*-thiolation of cPKα inhibits the activity of cPKα and its isozymes, while cysteine *S*-thiolation does not cause any change in the activity [70]. A chemical biology approach such as the utilization of Gsp may thus be an attractive option.

**Figure 7:**
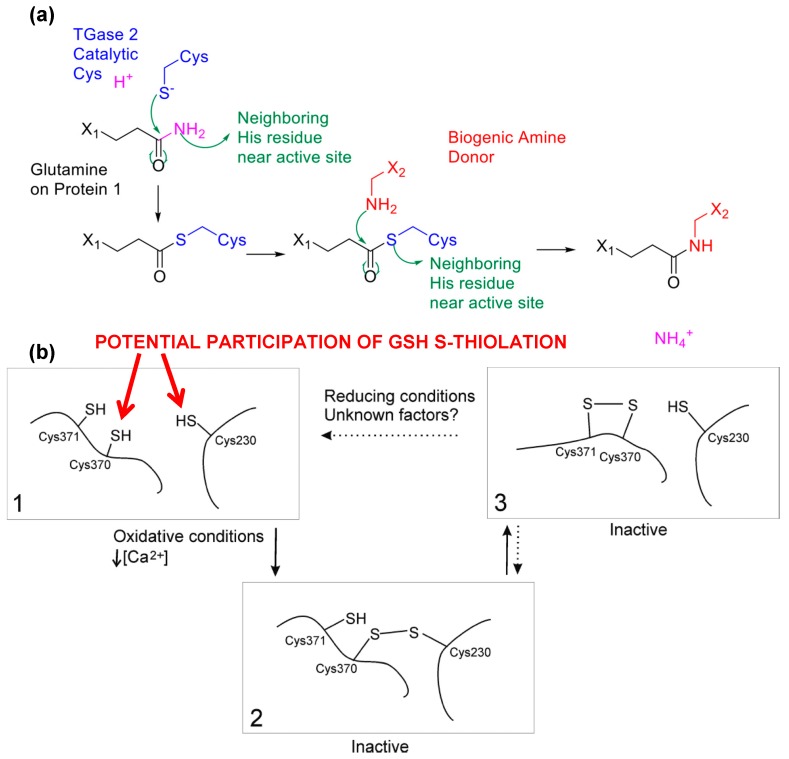
TGase 2 as a case study to demonstrate that Gsp is the key to understand the biochemistry of protein thiols. (**a**) Reaction mechanism of tissue transglutaminase upon activation is initiated by a nucleophilic attack by the activated catalytic cysteine on the glutamine amide. A second stage of nucleophilic attack from an amine donor transforms the thioester intermediate to complete the transamidation reaction; (**b**) A proposed scheme of the role of *S*-glutathionylation in TGase 2 activity moderation, as modified from [85]. *S*-glutathionylation at Cys 230 may promote disulfide formation in TGase 2 cysteine triads, providing energetically favorable incremental states to rapidly protect and activate TGase 2 in times of need. The generation of such intermediate states triggered by *S*-glutathionylation could lead to disulfide bridges among the triad and conformational changes in TGase 2 structure. Cysteine mapping results suggest that Cys 230 remains in potential disulfide bond formation independent of calcium, implying an equilibrium “resting” stage of *S*-glutathionylation to maintain TGase 2 for rapid activity. Figure 7b was originally published by Stamnaes *et al*. [85] © The American Society for Biochemistry and Molecular Biology.

The use of Gsp can also be extended to understand the biochemistry of a protein in particular by coupling mass spectrometric analysis. We here provide an example using tissue transglutaminase. Tissue transglutaminase (TGase 2) catalyzes the transamidation between a γ-carboxamide group of glutamine residues and an amine-containing molecule (e.g., ε-amine of lysine residue, Figure 7a). The enzyme has been found to be functionally related to apoptotic processes, and associated with various processes and phenomena such as serotonylation [71], G-protein-related functions (with its ability to bind and hydrolyze GTP), protein kinase and disulfide isomerase activities [72], the evasion of proteolysis in apoptosis [73], celiac disease [74], amyloidogenesis [75], neurodegenerative diseases [76,77] and even cancer [78,79,80].

The transamidation of spermine onto DNA-binding proteins such as histone or chromatin [81,82] also marks the significance of TGase 2 in cell development. The complexity of its biological function revolves around the mediation of oxidative stress, notably when cell defenses fail against the production of reactive oxygen species [83]. Nevertheless, conflicting literature on TGase 2 in stress responses [84] suggests that its biochemistry has not been fully understood, especially in relations to how TGase 2 is modulated in the presence of oxidative stress. As TGase 2 contains 20 cysteine residues, among which is a catalytic residue central to its function, the proper protection of those cysteine thiols is thus a critical issue for cells to maintain homeostasis. To date, there is little information on how TGase 2 is mediated by cysteine modifications beyond the possibility of modification at Cys 230 in a mutant TGase 2 [85]; while various studies infer that TGase 2 may be *S*-nitrosylated [86,87], there is insufficient residue-level evidence to confirm the true role of cysteine modifications on activity.

Our preliminary results based on mass spectrometric analysis described in [56] suggested four possible *S*-glutathionylated cysteine residues on TGase 2: cysteines 230, 370, 524 and 554, among which Cysteines 230 and 370 were previously implicated in TGase 2 activity [85,88] while the location of Cys 524 and Cys 554 in proximity to the nucleotide binding domain suggested the contribution to TGase 2’s signaling functions as a G-protein. Previously Cys 524 was suggested to participate in nitrosyl coupling [69], but the insufficient residue-level evidence meant that the actual mechanism remained unclear. When coupling the use of Gsp with other analytical methods such as differential thiol-trapping approaches, for instace OxICAT, one could further elucidate the complex biochemistry behind a particular enzyme of interest. Previously the inactivation of TGase 2 was found to be initiated by the formation of a Cys 230–370 disulfide followed by a subsequent and vicinal Cys 370–371 disulfide [85]. Curiously, some of our preliminary findings suggested that the catalytic residue (Cys 277) was not modified and independent of TGase 2’s activation status as previously hypothesized and observed [89]. This suggests that TGase 2’s activity changes must be primarily due to changes of other redox-sensitive cysteines, instead of simple protection-deprotection of the active site. Along the observation that the cysteine triad is in close proximity when TGase 2 is in a “closed” or “open” conformation [88,90], the stepwise formation of intramolecular disulfides among the cysteine triad must regulate the activation and inactivation of TGase 2 somewhat. Thus, modifications of cysteines 230 and 370 would suggest that intermediate states of inactivation facilitated by PSSG potentially promoted rapid and more energetically favorable disulfide formations at the cysteine triad (Figure 7b), in comparison to a simple two-state switch previously suggested. Cys 230 and 370 could potentially be first *S*-glutathionylated, and such a molecular event triggered the formation of a disulfide bond with the now reduced Cys 370 to further propagate the formation of a disulfide bond with Cys 370 and 371. This action placed TGase 2 in a “standby” state, which stabilized the enzyme until rapid oxidation was encountered.

Generally speaking, surface cysteines tend to be more likely to be exposed to reactive oxygen or nitrogen species, and subsequently become irreversibly oxidized, although previous findings and our preliminary results suggested otherwise. The modulation of oxidative stress, as it appeared, was tightly regulated on TGase 2. TGase 2 has been long thought to be a key regulatory factor in countering hypoxia, but the molecular mechanism is still unknown since there is insufficient molecular evidence connecting TGase 2 protein targets and signaling pathways to the intracellular modulation of oxidative stresses. Chemoenzymatic tools such as the use of glutathione derivatives like Gspm-biotin can provide useful insights and complementary evidence to questions only partially addressed by molecular biology.

## 6. Conclusions and Future Aspects

As this moment, aside from the possibility of niche survival in Enterobacteria and Kinetoplastids [24], little else is known about Gsp. Nevertheless, while its biological function remains a mystery, as a precursor to other small molecular thiols such as trypanothione, Gsp still serves as a promising target in drug discovery, particularly in the development of antiparasitic drugs. Trypanothione is a unique and essential redox metabolite of trypanosomatid parasites, and the synthesis of trypanothione occurs by the consecutive conjugation of two glutathione molecules to spermidine. Other low molecular weight thiols such as bacillithiol and mycothiols also can participate in cytosolic redox regulation. While most other low molecular thiols have been extensively studied [91,92,93,94], it is nonetheless important to note that these thiols are functionally analogous to glutathione and thus may be similarly characterized to reveal curious new insights. For instance, a recent study suggests that via chemical targeting of trypanothione synthetase with a drug-like compound could lead to parasite death [95], and the overall similarity of small molecular thiol synthetases suggest the possibility of wide-spectrum antiparasitic drugs based on such a target.

As cysteine is one of the most reactive and critical amino acid residues, the effects of undesired oxidation are also the most severe. Protective oxidation of such thiols via the mechanism of *S*-glutathionylation is thus an integral yet quite overlooked regulatory system *in vivo*. However, the lack of available tools does indeed hamper further investigations in this area. As the field of chemical biology continues to evolve, applications of seemingly unrelated tools can help answer difficult questions. While Gsp may be an exclusive feature of prokaryotic pathogens and have lost their usefulness evolutionarily in eukaryotes, the bifunctional nature of the corresponding synthetase can be applied to PSSG studies in a chemoenzymatic fashion. We discuss here potential uses of Gsp to observe the effect of protein *S*-thiolations in three aspects: profiling *S*-thiolated proteins in the human proteome in a site-specific manner, understanding the impact of *S*-thiolations on activity-related cysteine residues, and as a viable biochemical tool to characterize PSSG. Facilitated by efficient means of enrichment via the engineered tag, we could identify proteins through mass spectrometry-based proteomic analysis and also predict a mechanistic role of cysteine *S*-glutathionylation in TGase 2 activation.
